# A B-cell targeting virus disrupts potentially protective genomic methylation patterns in lymphoid tissue by increasing global 5-hydroxymethylcytosine levels

**DOI:** 10.1186/s13567-014-0108-5

**Published:** 2014-10-23

**Authors:** Nick A Ciccone, William Mwangi, Alexey Ruzov, Lorraine P Smith, Colin Butter, Venugopal Nair

**Affiliations:** Avian Viral Diseases (AVD), Compton Laboratory, The Pirbright Institute, Compton, Berkshire RG20 7NN UK; Wolfson Centre for Stem Cells, Tissue Engineering and Modelling (STEM), Division of Cancer and Stem Cells, School of Medicine, Centre for Biomolecular Sciences, University of Nottingham, University Park, Nottingham, NG7 2RD UK

## Abstract

The mechanisms by which viruses modulate the immune system include changes in host genomic methylation. 5-hydroxymethylcytosine (5hmC) is the catalytic product of the Tet (Ten-11 translocation) family of enzymes and may serve as an intermediate of DNA demethylation. Recent reports suggest that 5hmC may confer consequences on cellular events including the pathogenesis of disease; in order to explore this possibility further we investigated both 5-methylcytosine (5mC) and 5hmC levels in healthy and diseased chicken bursas of Fabricius. We discovered that embryonic B-cells have high 5mC content while 5hmC decreases during bursa development. We propose that a high 5mC level protects from the mutagenic activity of the B-cell antibody diversifying enzyme activation induced deaminase (AID). In support of this view, AID mRNA increases significantly within the developing bursa from embryonic to post hatch stages while mRNAs that encode Tet family members 1 and 2 reduce over the same period. Moreover, our data revealed that infectious bursal disease virus (IBDV) disrupts this genomic methylation pattern causing a global increase in 5hmC levels in a mechanism that may involve increased Tet 1 and 2 mRNAs. To our knowledge this is the first time that a viral infection has been observed to cause global increases in genomic 5hmC within infected host tissues, underlining a mechanism that may involve the induction of B-cell genomic instability and cell death to facilitate viral egress.

## Introduction

Cytosines within the genome not only constitute part of the genetic code but are also amenable to chemical modification making them a central conveyer of epigenetic information. Methylation of the fifth position of cytosine (5-methylcytosine, 5mC) is an evolutionarily conserved epigenetic modification [1] which helps to maintain genome stability and acts as a suppressive mark for gene expression [2]. It is becoming apparent that genomic DNA demethylation is more prevalent and dynamic than was previously appreciated. A mechanistic understanding of active DNA demethylation indicates the involvement of cytosine hydroxymethylation (5hmC) [3]. The Tet (Ten-11 translocation) proteins can convert 5mC to 5hmC [3,4] making these enzymes pivotal players in events leading to complete cytosine demethylation [5]. The finding that many tissues accumulate substantial 5hmC levels [6,7] allows for the intriguing possibility that this cytosine modification is not only a transient intermediate leading to complete DNA demethylation but may also be an epigenetic entity that carries its own unique coding properties and consequences.

Although the biological role of 5hmC and Tet proteins remain to be fully established, current models suggest their involvement in vertebrate embryonic development [8-10], while the abundance of 5hmC within gene bodies and enhancers has been ascribed to a role in modulating transcription [6,11]. Recent studies have observed global genomic increases in 5hmC in somatic tissue during aging [12] and as a characteristic feature of disease [13]. In contrast, disruptions to Tet 1 and 2 functions have been associated with reduced 5hmC levels in various forms of cancer [7,14]. Such examples include leukaemia which is often associated with mutations in the catalytic activity of Tet 2 leading to diminished 5hmC levels in hematopoietic stem cells (HSC), delayed HSC differentiation and skewed development toward a monocyte/macrophage lineage [15,16]. Collectively, these studies suggest that disruption to the correct regulation of genomic 5hmC is not only a diagnostic marker for disease but also suggests that changes in 5hmC levels may be part of a causal mechanism underlying the pathogenesis of multiple disorders including those of the immune system.

Viruses, being obligate intracellular parasites, have evolved several sophisticated mechanisms to hijack cellular machinery and to evade their host’s immune system. Oncogenic viruses, including those that infect immune cells, are known to modulate the expression of DNA methyltransferases to silence tumor suppressor genes through promoter hypermethylation [17-20]. To our knowledge, no study has explored possible changes in host genomic 5hmC levels after viral infection.

As vertebrates whose embryonic stages are readily accessible to investigation, chickens have made major contributions to many areas of immunology and development [21]. It was in chickens that the existence of the bursa of Fabricius (BF) and the B-cells that specialise in antibody production within it was first described [21]. Avian B-cells are crucial for inducing antibody responses against viral pathogens; in response viruses that infect birds often target B-cells to avoid elimination [22,23]. One such pathogen of veterinary and economic significance is infectious bursal disease virus (IBDV) which infects and subsequently causes cell death of developing B-cells as part of a mechanism to avoid elimination from the host [23-25]. Chickens within a commercially produced flock can recover from IBDV infection but remain immunosuppressed due, in part, to a reduced B-cell capacity. These birds are susceptible to future pathogen infections and respond poorly to vaccination regimes to protect flocks from other viral infections of economic importance [23,24]. In the current study we investigated possible changes in 5hmC and 5mC patterns during the ontogeny of B-cells within the developing BF and tested the hypothesis that IBDV infection disrupts the genomic methylation patterns within this primary lymphoid organ.

## Materials and methods

### Animals and IBDV infection

Specific pathogen free (SPF), inbred line 0 or 15 L chickens and either fertilized Rhode Island Red eggs containing viable embryos or neonatal chicks were obtained from the poultry production unit at The Pirbright Institute. Both rearing and experimentations were conducted in accordance with the UK animal scientific procedures act 1986 and were approved by the Pirbright Institute internal ethical review procedure and were performed under Home Office guidelines of the United Kingdom. For experiments concerning the developing bursa of Fabricius, chicks at various embryonic stages of development or post hatch were culled and bursa were rapidly removed and stored until tissue analysis (see relevant method heading for further information). For IBDV studies, an infectious dose of virulent UK661 IBDV strain or phosphate buffered saline (PBS) alone was administered per nares in a fifty microliter volume to six-week old chickens. Birds were culled 48 hours post infection (hpi) and bursa were rapidly dissected and processed.

### Immunofluorescence and bioimaging

Bursal tissue were sectioned using a cryostat (Leica CM1900, Milton Keynes, UK) set at a cutting width of 0.6 μM and subsequently fixed in 4% paraformaldehyde for thirty minutes at ambient temperature. Sections were then permeabilized in 1% Triton X100 (Sigma-Aldrich, Poole, UK) for 15 min at room temperature before incubation at 37 °C in either 2N or 4N HCl (Sigma-Aldrich) for one hour. Primary antibodies were diluted in 10% fetal bovine serum (FBS) and incubated for one hour at room temperature. To detect specific genomic cytosine modifications, antibodies raised against 5hmC (Active Motif, La Hulpe, Belgium, 1:1000) and 5mC (33D3, Diagenode, Seraing, Belgium, 1:500) were used. While a monoclonal antibody (AV20, Southern Biotech, Birmingham, AL, USA) directed against the Bu-1 molecule was used to specifically detect B-cells. Secondary fluorescent antibodies, Alexa 568 goat anti-rabbit (Life Technologies, Paisley, UK) to visualize 5hmC and either Alexa 488 goat anti-mouse (Life Technologies) to detect 5mC or Bu-1, were diluted 1:500 in 10% FBS and incubated at room temperature for one hour. After washing in PBS, sections were either incubated in 4′, 6-Diamidino-2-phenyindole (DAPI; Sigma-Aldrich) diluted 1:10 000 in dH_2_O for 10 min at room temperature for cellular DNA conterstaining prior to mounting or mounted directly in mounting media (Vector Laboratories, Peterborough, UK). Control staining without primary antibody incubation gave either no or minimal detectable signal. Images were captured using a Leica TCS SP5 confocal microscope linked to a personal computer running LAS AF software (Leica Microsystems, Milton Keynes, UK).

### DNA dot blot assay

Bursal tissue were placed in 2.0 mL screw cap tube containing 10 mm glass beads (Biospec products) in proteinase K buffer (10 mM Tris, pH8.0, 100 mM NaCl, 10 mM EDTA, 0.5% SDS) and disrupted in an automated tissue homogenizer (Biddy Scientific, Stone, UK) for thirty seconds. The solution was transferred into a clean tube and 100 μg proteinase K (Sigma-Aldrich) was added and incubated at 50 °C in a hybridization oven (Hybaid, Basingstoke, UK) with end over end rotation overnight or until tissue had dissolved. Samples were spun at 6000 rpm in a table microfuge and the supernatant was transferred to a new tube. DNA was phenol-chloroform extracted using standard protocols. Purified DNA was quantified and 1 μg of DNA from each sample was serially diluted 1:10 and denatured in 0.4M NaOH at 100 °C for 10 min. Samples, neutralized with an equal volume of chilled Tris–HCl (pH 6.8) were applied to a nitrocellulose membrane (GE Healthcare, Hatfield, UK). DNA was then hybridized by ultraviolet crosslinking in a UV stratalinker 1800 (Stratagene, Cambridge, UK) and the membrane was incubated at room temperature in 3% non-fat Milk (Bio-Rad, Hemel Hempstead, UK) TBS-T containing either a 1:5000 dilution of anti-5hmC (Active Motif), 1:2000 dilution of anti-5mC (33D3, Diagenode) for two hours with shaking. Appropriate secondary antibodies (HRP conjugated donkey anti-rabbit or HRP conjugated donkey anti-mouse, Cell Signalling, Hitchin, UK) were diluted 1:5000 in 3% non-fat Milk in TBS-Tween 20 and blots were incubated with agitation for one hour. Blots were visualized using ECL solution (Millipore, Watford, UK) and exposed to X-ray film (GE Healthcare).

### Quantitative reverse transcriptase real-time PCR (qRT-PCR) assay

Bursal tissues were placed in RNA later (Qiagen, Manchester, UK) immediately after dissection. At the time of extraction, tissues were cut into a size that weighed ~30 mg and disrupted using glass beads (Biospec, Glasgow, UK) and an automated tissue homogenizer (Biddy Scientific). Lysates were spun through a QIAshredder (Qiagen) before RNA extraction using a RNeasy kit (Qiagen) and 500 ng of total RNA was reverse transcribed using superscript III (Life Technologies). One microliter of cDNA was mixed with platinum SYBR green reagent (Life Technologies) and used in a qRT-PCR assay, performed on an ABI 7900 96-well real-time thermocycler, to quantify relative amounts of cytosine modifying enzymes Tet 1, Tet 2 and activation induced deaminase (AID) transcripts. Glyceraldehyde-3-phosphate dehydrogenase (GAPDH) was used as a reference gene and the ΔΔCt method of quantification was performed to obtain relative fold change to either embryonic day (ED) 16 bursa or from uninfected six-week old bursa. Primer sequences for AID (forward: CCTGCGTAACAAGATGGGTTGCCATGTGGAG and reverse: CGGGCAGTGAAAATGCGGAGGGTCAAGT) and Glyceraldehyde 3-phosphate dehydrogenase (GAPDH) (forward: GAAGCTTACTGGAATGGCTTTCC and reverse: CGGCAGGTCAGGTCAGGTCAACAA) were published previously and validated to work efficiently in real time qPCR assays. Probes directed against chicken Tet 1 (forward: AAAAGGAAGCGCTGTGAGAA and reverse: CCACGCCAGTATGAGAATCA) and Tet2 (forward: CGGTCCTAATGTGGCAGCTA and reverse: TGCCTTCTTTCCCAGTGTAGA) were designed based on sequences from Ensembl (ENSGALG00000004073.4 and ENSGALT00000017236 respectively). Statistical analysis, either a two way ANOVA followed by Bonferroni *post hoc* test or unpaired *t*-test were used to determine statistical significance between experimental groups using Prism 5 (Graphpad Software).

### Western blot

Approximately twenty micrograms of tissue were cut into fragments and placed in 2.0 mL screw cap tube containing 10 mm glass beads (Biospec) in 500 μL of RIPA buffer (Sigma) supplemented with protease inhibitors (Roche, Burgess Hill, UK) were disrupted using an automated tissue homogenizer (Biddy Scientific) for two thirty second pulses. Supernatant was transferred to a clean tube and spun at 14 000 rpm in a refrigerated table top microcentrifuge. Twenty microliters of the supernatant was run on a 12% SDS-PAGE gel and transferred to a nitrocellulose membrane (GE Healthcare) using a semi-dry transfer unit (Bio-Rad). The blot was blocked in 3% non-fat milk at ambient temperature for one hour on a rotating shaker before incubation with an antibody directed to the IBDV capsid protein VP2 (generated by the microbiological services of The Pirbright Institute) in a 1:500 dilution overnight at 5 °C with gentle agitation. The blot was rinsed and a secondary HRP conjugated anti-mouse antibody (Sigma-Aldrich) was used at 1:10 000 dilution for one hour at room temperature before rinsing and visualization using ECL solution (Millipore) and exposure to X-ray film (GE Healthcare).

## Results

### Changes in 5mC, 5hmC and mRNAs of cytosine modifying enzymes during development of B-cells within the bursa of Fabricius

**Figure 1:**
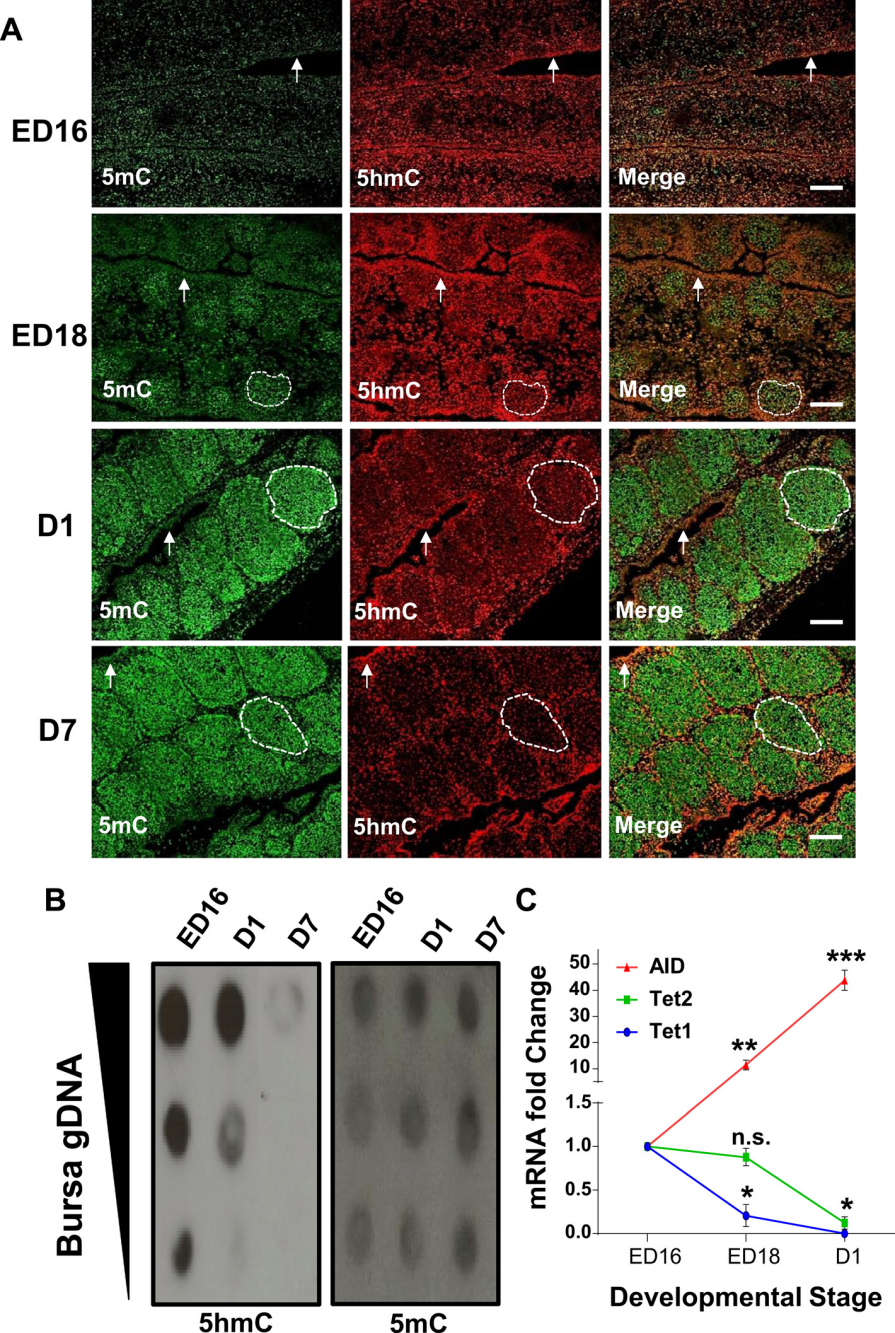
**Changes in global 5hmC and 5mC levels and gene expression of cytosine modifying enzymes within the bursa of Fabricius during embryonic and neonatal development. A**. Immunofluorescent staining of sections through embryonic day (ED) 16, ED18, one (D1) and seven days post hatch (D7) bursa of Fabricius. White dashed outlines delineate an individual follicle and arrows indicate epithelial structures. Scale bar = 100 μM. **B**. Dot blot assay using ten-fold dilutions of bursa genomic DNA from various developmental stages probed with an antibody directed to either 5hmC or 5mC. **C**. Real time analysis of cytosine modifiying enzymes AID, Tet 1 and Tet 2 mRNA expression levels during the development of the bursa of Fabricus. n.s. =non-significant, *p < 0.05, **p < 0.01, ***p < 0.005 as determined by ANOVA and *post-hoc* tests. *n* = 4-5 birds per group.

Using immunofluorescence we investigated potential changes in bursal 5mC and 5hmC genomic distribution at different embryonic and neonatal stages that encompass hallmarks of B-cell development. At embryonic day (ED) 16, the bursa had a relatively uniform distribution of both 5mC and 5hmC, although more intense 5hmC staining was consistently observed within the epithelial layers of the bursa (Figure 1A; arrows). This 5hmC enrichment becomes more apparent at successive stages of embryonic development when individual follicles can be definitively identified (dashed white outline) and become surrounded by a follicle associated epithelium (Figure 1A).

**Figure 2:**
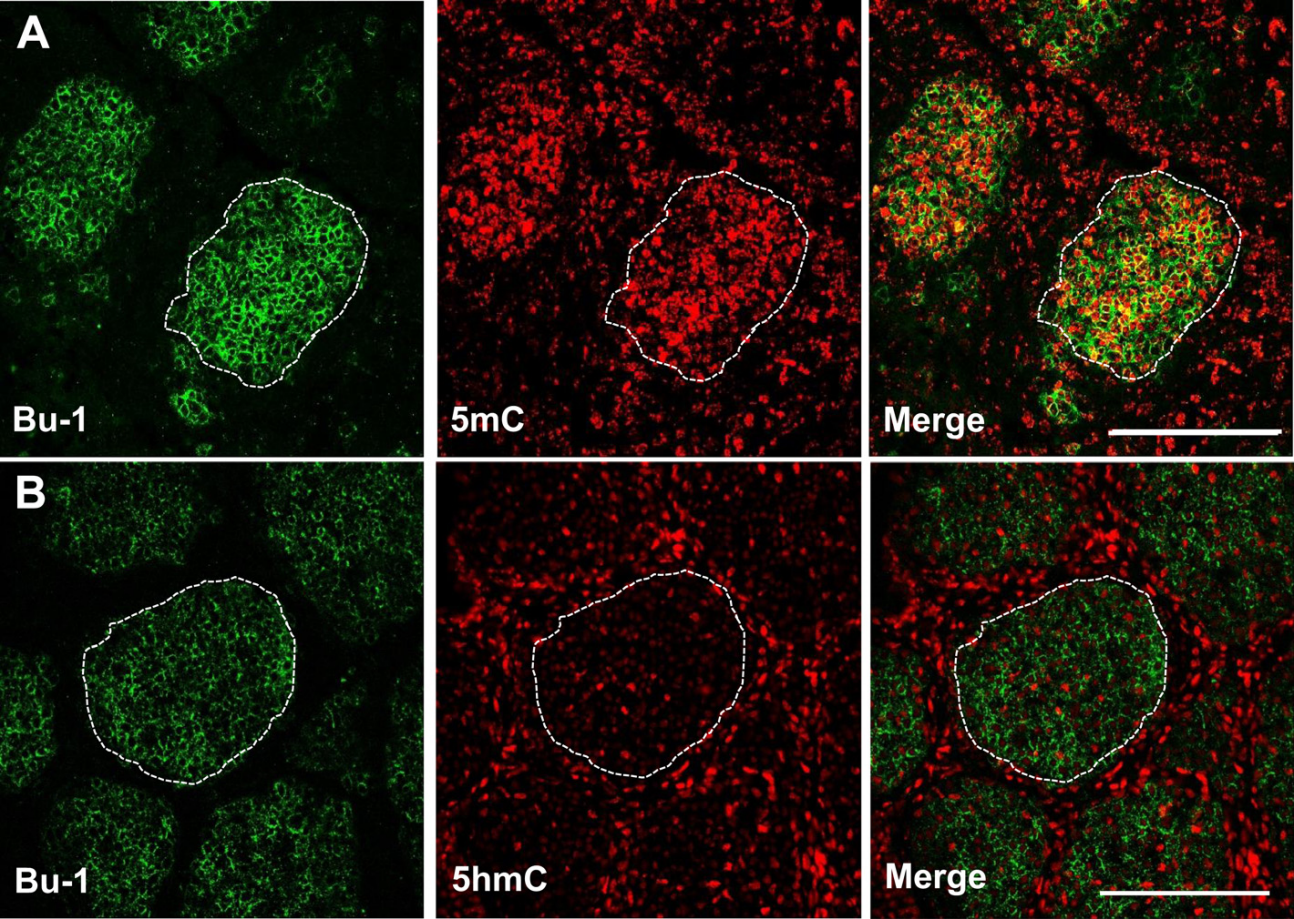
**The distribution of 5hmC and 5mC relative to B-cell localisation within the embryonic bursa of Fabricius.** Immunofluorescent staining of bursal sections of an embryonic day (ED) 18 chick showing the co-localisation of either **A**. 5mC or **B**. 5hmC with the avian B-cell marker, Bu-1. Dashed outlined area delineates a single B-cell containing bursal follicle. Bar = 100 μM.

Associated with high 5mC levels in developing follicles (Figure 1A; dashed white outline) is a progressive developmental loss of 5hmC staining. This observed 5hmC reduction in bursal tissue sections was validated by a dot blot assay (Figure 1B) which shows a progressive reduction in genomic 5hmC from ED16 to seven days post-hatch (D7). To confirm that the proliferating follicles did contain B-cells we co-stained ED18 bursal sections with Bu-1, an avian B-cell marker [26], and either an antibody directed to 5mC or 5hmC. We found that Bu-1 only stained cells within each follicle and the majority of these B-cells were also highly enriched for 5mC (Figure 2A). Furthermore, we observed that the B-cell genome contained relatively low levels of 5hmC compared to the surrounding bursal epithelial layers (Figure 2B).

With the increasingly 5mC enriched B-cell genome is the accumulation of bursal AID transcripts which increases approximately ten fold from ED16 to ED18 and forty fold from ED16 to one day post hatch (Figure 1C). While mRNA expression of both putative Tet 1 and 2 in the developing bursa significantly decreased from one day post hatch when compared to the bursa of ED16 chicks (Figure 1C).

### Effects of IBDV on 5hmC levels and Tet 1 and 2 mRNAs levels within infected bursa

**Figure 3:**
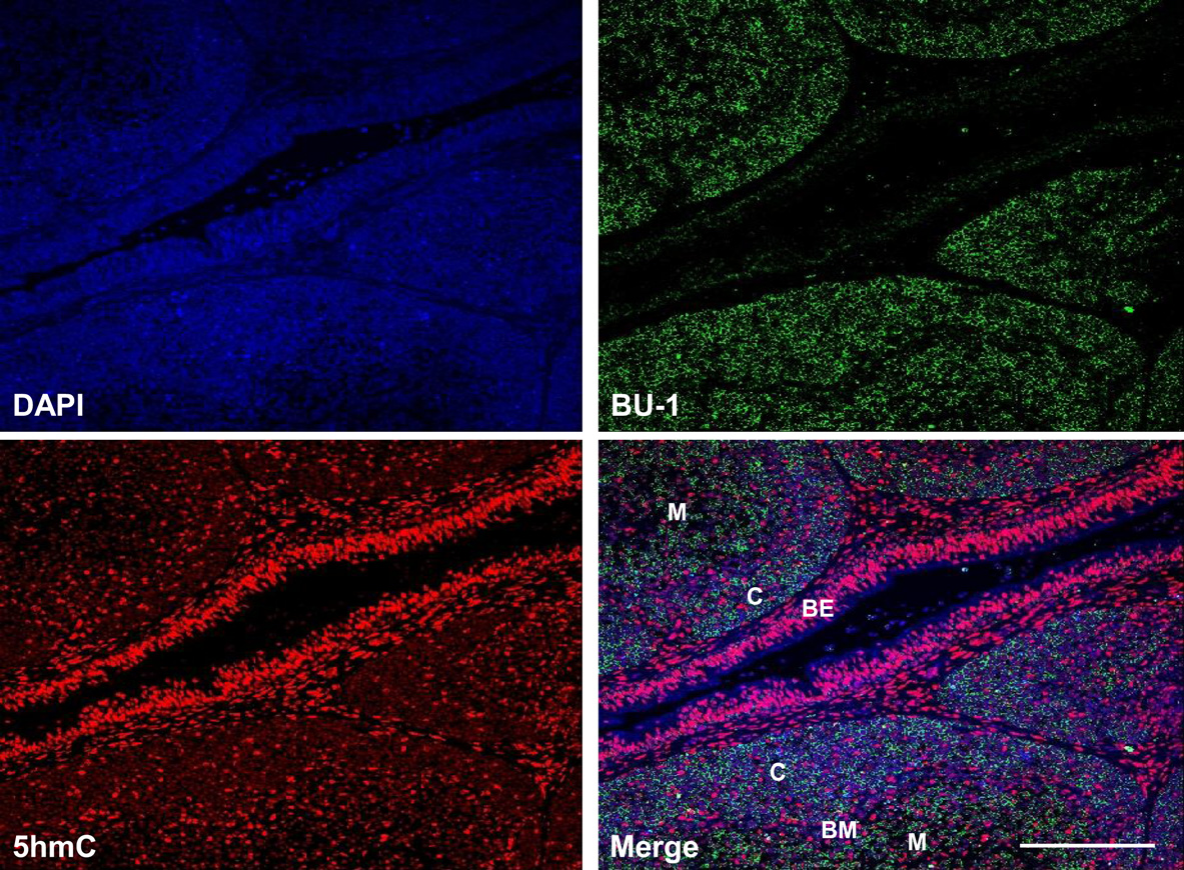
**Evidence suggesting that 5hmC is enriched within bursal epithelia compared to B-cells.** The bursa of a six week old chicken stained with 5hmC and the avian B-cell marker, Bu-1. DAPI used for counterstaining double stranded DNA. M = Medulla, C = cortex, BE = bursal epithelium, BM = basement membrane. Bar = 100 μM.

**Figure 4:**
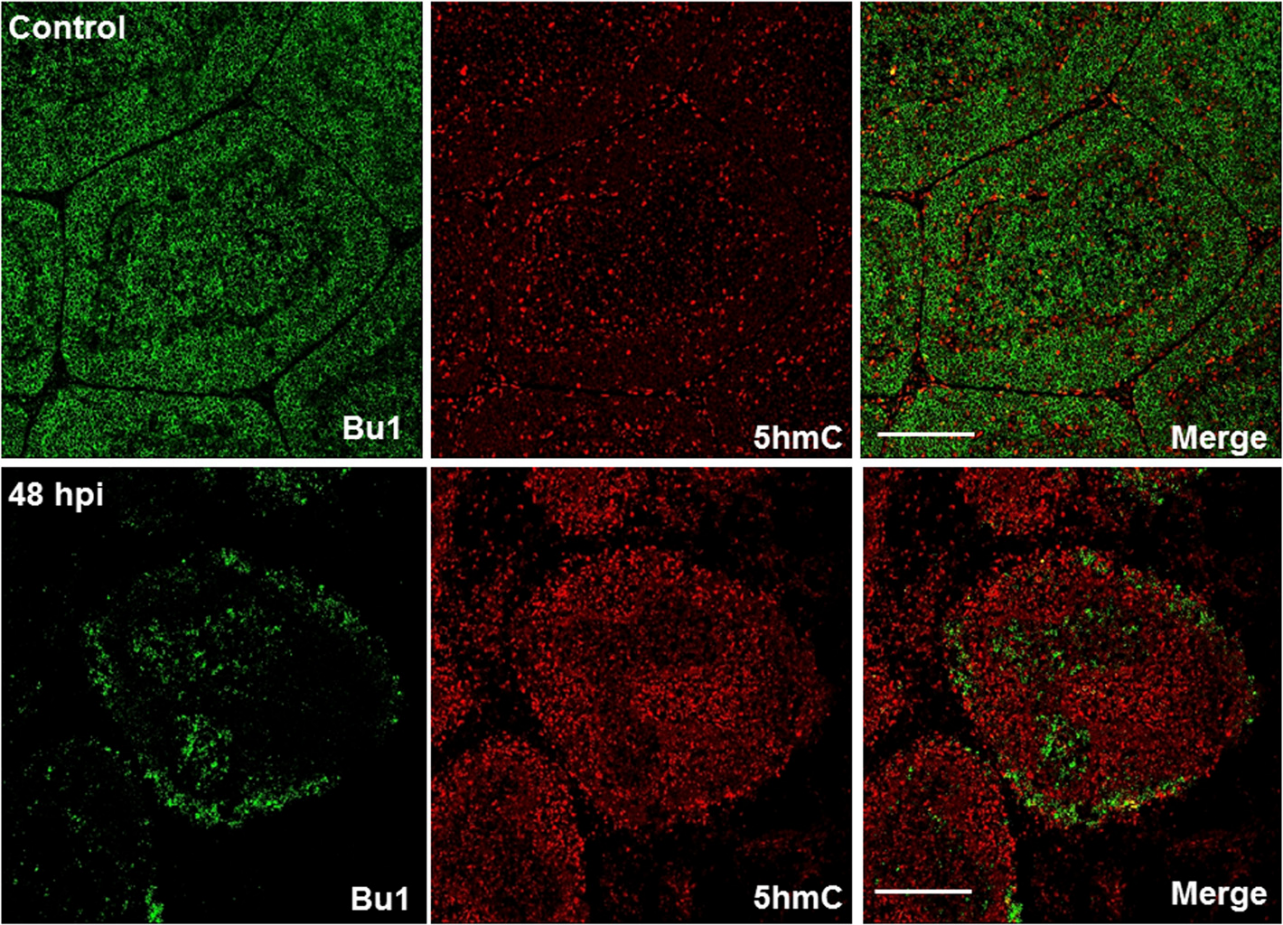
**Changes in Bu-1 and 5hmC within the bursa of Fabricius between control and after IBDV infection.** Immunofluorescent staining of bursal sections from either control or IBDV infected (48 hpi) 6-week old chickens showing co-localisation of 5hmC with the avian B-cell marker, Bu-1. M = Medulla; C = Cortex. Bar = 100 μM.

After hatch the bursa goes through a series of morphological changes which culminate in the formation of distinct cortical and medullary regions delineated by a basement membrane (BM) (Figure 3). We stained bursa from a six-week old chicken with antibodies to Bu-1 and 5hmC. We found that the majority of bursal cells were Bu-1 positive (Figures 3 and 4A). Less intense staining for Bu-1 was consistent with the location of the BM and bursal epithelium (BE), while 5hmC staining appeared more intense within cells that were not Bu-1 positive such as within the BM and the BE (Figure 3).

**Figure 5:**
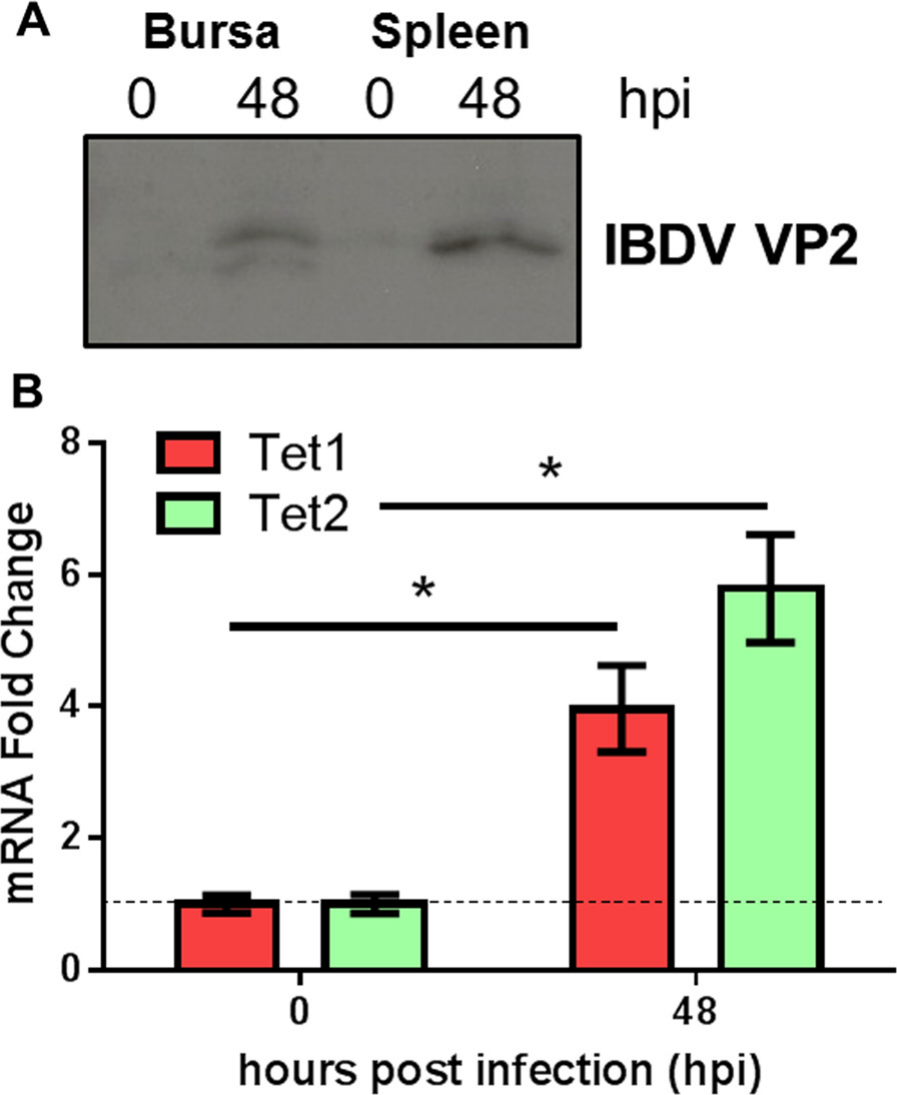
**Tet 1 and 2 mRNAs increase after 48 hpi of IBDV within the bursa of Fabricius. A**. Western blot analysis indicating that the capsid protein of IBDV, VP2, is present in both bursa and spleen of six-week old chickens infected with IBDV (48 hpi) but not in uninfected control birds. **B**. Relative expression of Tet 1 and Tet 2 mRNAs following IBDV infection (48 hpi) within the bursa of Fabricius. Bars are mean ± SEM. *Indicates statistical significance at the *p* < 0.05 level between uninfected and 48 hpi birds for the same gene as determined by unpaired *t*-test. *n* =3 birds per group.

Infection of six-week-old chickens with a dose of a virulent IBDV strain, UK661, caused a significant reduction in Bu-1 staining within the bursa after 48 hpi (Figure 4). Associated with this loss of Bu-1 was a global increase in immunostaining of 5hmC levels within the infected bursa when compared to uninfected, age-matched controls (Figure 4). Analysis of Tet 1 and 2 mRNAs by real time PCR revealed a significant increase in both transcripts within the bursa after IBDV infection (48 hpi) when compared to uninfected birds (Figure 5).

## Discussion

Infectious bursal disease is a contagious viral induced inflammatory condition of economic and veterinary importance that effects commercial poultry flocks [23,24]. IBDV targets cells of the BF where the virus predominantly focuses on infecting actively proliferating and differentiating lymphocytes of the B-cell lineage of the chicken [25]. Reports have documented global genomic changes in methylation patterns, including the novel epigenetic mark 5hmC, during both hallmarks of vertebrate development [9] and the pathogenesis of cancers like leukaemia [15,16] and inflammatory diseases such as osteoarthritis [27]. In light of these findings, and given the predilection of IBDV for the BF, we investigated potential changes in genomic methylation during key stages of B-cell development and after IBDV infection.

Although the BF plays a critical role in avian B-cell development the commitment to the B-cell lineage occurs within embryonic hemopoietic stem cells outside this gut associated lymphoid organ [28]. The BF is subsequently colonized between eight and fourteen days of embryonic development by these prebursal stem cells where they expand extensively to form follicles [28]. Discernable follicle like structures could be determined by 5mC and 5hmC staining in bursal sections at around ED18 (Figure 1A). These follicles were enriched for 5mC staining when compared to the surrounding epithelium, a feature that is maintained through both embryonic and post hatch stages of BF development. We determined that these follicular cells were B-cells by co-staining with Bu-1, an avian B-cell marker (Figure 2A). A possible reason for why developing B-cells have a predominantly high genomic 5mC level is due to the consequences of gene conversion, the process responsible for generating the primary antibody repertoire in the majority of vertebrates [29]. Gene conversion is dependent on the cytosine modifying enzyme activation-induced deaminase (AID) which generates antibody diversity at immunogloblin loci through targeted deamination of cytosines [29]. This process begins within the bursa at around ED18 [30,31] and the highly methylated B-cell genome may protect from the mutagenic activity of AID at off target sites as this enzyme is inefficient at deaminating 5mC, particularly in the context of CpG motifs [32,33]. The observation that AID is expressed in the bursa is consistent with both previous studies [34] and its involvement in antibody diversification by gene conversion in vivo, further supporting the notion that progressive methylation may protect the integrity of the B-cell genome from the increased expression of this deaminating enzyme (Figure 1C). These findings coincide with the progressive decrease in bursal 5hmC levels, as measured by two independent techniques (Figure 1A and B), and suggests that the reduction in Tet mRNA transcripts (Figure 1C) maybe causal to reduced genomic 5hmC within the developing BF. As mammalian Tet and AID proteins have been shown to interact [35] it is conceivable that Tet proteins may facilitate AID binding to genomic regions within B-cells. In this way, a developmental reduction in Tet 1 and 2 mRNAs may be part of a mechanism by which the B-cell genome is protected from the mutagenic activity of AID by reducing such interactions that could lead to off target genomic mutations.

As Bu-1 is reduced considerably after IBDV infection within the BF (Figure 4) it remains unclear what cell type has elevated 5hmC within this tissue. It is possible that IBDV downregulates Bu-1 expression in B-cells as is the case for reticuloendotheliosis virus T (V. Nair, unpublished observation), in this way, the increase in 5hmC may pertain to Bu-1 negative B-cells infected with IBDV in a mechanism that is likely to involve increased Tet 1 and 2 mRNAs (Figure 5B). After an IBDV infection macrophages and T-cells are known to infiltrate the BF [23,36] and it is conceivable that these immune cell types are contributing to the observed increase in bursal 5hmC levels and Tet 1 and 2 mRNAs. In support of this view is the finding that both activation of macrophages and T-cells causes genomic demethylation events which may include the involvement of elevated 5hmC levels and altered Tet function [37]. Furthermore, there is an emerging concept that pro-inflammatory cytokines may modulate Tet expression and 5hmC levels to cause transcriptional changes in cytokine responsive genes [27]. In this way, the release of cytokines from immune cells that have infiltrated the bursa may contribute to altered 5hmC levels observed in the current study and may be causal to genomic instability and subsequent cell death in B-cells. Alternatively, or in addition, elements of the bursal stromal network including the BM and BE (Figure 3) may also be responding to IBDV infection to mediate the documented changes within the BF. The identity of which cell types are contributing to elevated 5hmC and Tet mRNA expression would be of interest and will form the bases for future studies.

In summary, we find that global 5hmC and 5mC genomic levels display temporal changes during the development of B-cells within the BF. Moreover, we show that the chicken embryonic B-cell has high levels of genomic methylation that may protect from the disruptive activity of the cytosine modifying enzyme, AID. IBDV infection causes immunosuppression through loss of B-cells and our data suggest that the underlying mechanisms may include disruption to genomic methylation patterns of the BF and an increase in global 5hmC genomic levels. This increase in 5hmC within infected host tissues may be part of a hitherto unrecognised mechanism by which a virus can cause genomic instability and cell death to facilitate viral egress.

## Abbreviations

5hmC: 5-hydroxymethylcytosine
5mC: 5-methylcytosine
AID: Activation-induced deaminase
Tet: Ten-11 translocation
hpi: Hours post infection
ED: Embryonic day
IBDV: Infectious bursal disease virus
BF: Bursa of Fabricius
BM: Basement membrane
BE: Basement epithelium
